# Quantifying the link between art and property prices in urban neighbourhoods

**DOI:** 10.1098/rsos.160146

**Published:** 2016-04-27

**Authors:** Chanuki Illushka Seresinhe, Tobias Preis, Helen Susannah Moat

**Affiliations:** Data Science Laboratory, Behavioural Science, Warwick Business School, University of Warwick, Coventry CV4 7AL, UK

**Keywords:** urban economics, urban gentrification, art, online data, data science, computational social science

## Abstract

Is there an association between art and changes in the economic conditions of urban neighbourhoods? While the popular media and policymakers commonly believe this to be the case, quantitative evidence remains lacking. Here, we use metadata of geotagged photographs uploaded to the popular image-sharing platform *Flickr* to quantify the presence of art in London neighbourhoods. We estimate the presence of art in neighbourhoods by determining the proportion of *Flickr* photographs which have the word ‘art’ attached. We compare this with the relative gain in residential property prices for each Inner London neighbourhood. We find that neighbourhoods which have a higher proportion of ‘art’ photographs also have greater relative gains in property prices. Our findings demonstrate how online data can be used to quantify aspects of the visual environment at scale and reveal new connections between the visual environment and crucial socio-economic measurements.

## 1. Background

The story of art playing a central role in the transformation of deprived urban neighbourhoods is a compelling one that has greatly engaged popular media and policymakers alike. Researchers of urban policy maintain that the creative industries are key to building a successful city economy [1,2]. Governments around the world make significant investments in the arts in order to incentivize gentrification and regeneration in specific neighbourhoods [3–5].

However, quantitative evidence linking the presence of art with changing economic conditions of urban neighbourhoods is lacking. Several studies focus on specific neighbourhoods that fit this narrative, rather than carrying out broader investigations to determine whether this pattern holds in general [6–10]. Other studies use broad definitions to examine the role that artists or creative people play in the economic improvement of urban neighbourhoods, thereby including groups of people with differing agendas [11]. For example, studies often include professions such as engineering, mathematics and computer science [2,12], or categories such as ‘Architectural Services’ and ‘Dance Companies’ [13]. Other research attempting to use art galleries and arts organizations to quantify arts-led redevelopment in urban neighbourhoods suggests that once art institutions have moved into a neighbourhood, redevelopment is already taking place [14,15] or a high level of income is already present [16].

A key challenge for studies to date has been the identification of a reliable metric that quantifies the presence of art in an area. Traditional studies have relied upon categories of creative occupations [2,12,13] or the presence of art institutions [14–16], which are potentially constraining measures that may not accurately capture the level of art present in a particular area.

Online activity could help us trace the presence of art in neighbourhoods in a less restrictive fashion. Today, we have access to a new source of information on human behaviour: patterns of activity on platforms such as *Google*, *Flickr*, *Twitter* and *Wikipedia*, which have already led to several insights into real-world human behaviour [17–32]. Specifically, such studies demonstrate that we can use these new data sources to quantify aspects of human behaviour that have previously been too costly, time-consuming or awkward to measure. For example, Seresinhe, Preis and Moat found a link between scenic environments and people's wellbeing [30], by analysing novel crowdsourced data garnered through an online game called *Scenic-or-Not*, alongside data from the Census for England and Wales. Using this online game, over 1.5 million ratings of images covering 95% of Great Britain at a granularity of 1 km^2^ have already been collected.

Here, we propose to create an indicator of the presence of art by exploiting photographs uploaded to the popular image sharing website *Flickr.* We focus on London as our area of study, as artists have long thought to be changing the economic landscape of London—from James Whistler and Oscar Wilde in Chelsea, to the 1990s ‘Britart’ movement in Hoxton [33].

## 2. Material and methods

We draw on images uploaded to *Flickr* between 2004, the year *Flickr* was released to the public, and 2013. As we are primarily interested in observing the presence of art in urban areas, we only include images geotagged as being located in Inner London.

In order to identify and separate areas within Inner London for analysis, we use the system of postcodes maintained by the Royal Mail. Postcodes are alphanumeric references comprising an ‘outward code’ of two to four characters and an ‘inward code’ of three characters (e.g. CV4 7AL). Postcodes beginning with NW, N, W, WC, E, EC, SW and SE are deemed as being located in Inner London. As the inward code is specific to a single section of a street, we only use the outward code of the postcode (e.g. ‘EC1’)—the postcode district—to locate neighbourhoods in Inner London. To ensure consistency between the size of postcode areas, in the EC and WC areas, we aggregate the postcode districts by ignoring the further subdivision created by the additional letter in the outward code. For example, the postcodes ‘EC1A’ and ‘EC1M’ are considered part of the area ‘EC1’, and data for these postcodes are therefore, analysed together.

When photographers upload images to *Flickr,* they can include metadata such as a title, description and tags (e.g. ‘art’, ‘sky’, ‘modern’). We deem a photograph to be an ‘art’-related image if there is a mention of ‘art’ in this textual metadata associated with *Flickr* photographs for each Inner London postcode. In this analysis, we restrict ourselves to classifying images using textual data only. Future analysis could consider image recognition techniques to classify images as ‘art’-related [34], or could work with citizen scientists to determine whether images contain art, following the example of such initiatives as *Galaxy Zoo* where volunteers classify images of galaxies [35].

In order to create an indicator of ‘art’-related images, we search all the textual elements of each image, including the title, description and tags, using the regular expression ‘<art>‘, which ensures that only the whole word ‘art’ is found. We note that in regular expressions in R, the symbols ‘<‘ and ‘>‘ match the empty string at the beginning and end of a word. While this process ensures we capture the word ‘art’ and no other words that contain ‘art’ (such as ‘apart’), this method does have a minor drawback in that words such as ‘arty’ or art-related compound words that do not have spaces (such as ‘streetart’) are not discovered. We count only a single occurrence of ‘art’ for each image, even if it has ‘art’ mentioned several times in the metadata.

Certain areas in London will attract more images on *Flickr* than others (e.g. tourists generate many images of notable locations such as Big Ben and the London Eye). Therefore, in order to meaningfully compare one area with another, we divide the number of ‘art’ images by the total number of images for each postcode for the whole time period (2004–2013), in order to create a normalized indicator of ‘art’ per area.

We underline that with this approach, images that are in reality ‘art’ images but do not have the word ‘art’ in their textual metadata will not be identified. In addition, images which have the word ‘art’ in their metadata but do not in reality contain any art will count towards our indicator. Indeed, our indicator is susceptible to any outside influence which may cause photographers to upload a higher proportion of photographs with the word ‘art’ in the textual metadata—for example, the presence of more photographers who are interested in art, rather than an increased presence of art.

**Figure 1.**
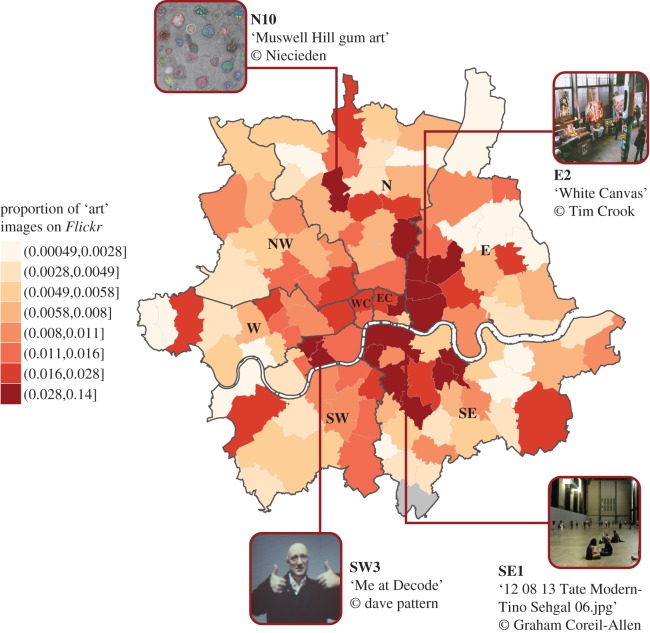
Proportion of ‘art’ photographs uploaded to *Flickr* from 2004 to 2013 in Inner London postcode areas*.* We deem a photograph as ‘art’ related if there is a mention of ‘art’ in the photograph's textual metadata. As tourist areas in London will attract more images on *Flickr* than other areas, we divide the number of ‘art’ images by the total number of images for each postcode for the whole time period (2004–2013), in order to calculate the proportion of ‘art’ photographs per Inner London neighbourhood. We define neighbourhoods on the basis of postcodes, as explained in detail in the main text. With this measure, we find that the proportion of ‘art’ related photographs is highest in East and Southeast London, with some heightened activity in Southwest and North London. All photos from *Flickr* are licensed for re-use under the Creative Commons Attribution-Non-Commercial-ShareAlike 2.0 Generic Licence (https://creativecommons.org/licenses/by-nc-sa/2.0/). Postal Boundaries © GeoLytix copyright and database right 2012. Contains Ordnance Survey data © Crown copyright and database right 2012. Contains Royal Mail data © Royal Mail copyright and database right 2012. Contains National Statistics data © Crown copyright and database right 2012.

With these limitations in mind, our indicator allows us to estimate which neighbourhoods in London may have relatively more ‘art’, or photographers interested in art, over this time period. Visual inspection of the proportion of ‘art’ images in Inner London postcodes reveals that art is most prevalent in East and Southeast London, with some sparse heightened activity in Southwest, North and East Central (figure 1).

Next, as one indicator of the economic transformation of Inner London neighbourhoods, we use a measure of the relative change in mean residential property prices. We track residential property prices in Inner London using data from residential property sales registered at the Land Registry from 2004 to 2013 (https://www.gov.uk/government/collections/price-paid-data). To understand the relative increase in house price in comparison with other areas, we first calculate the mean residential property price per Inner London postcode. (Note that as residential property prices have only been recorded since 2013 for the newly created postcode ‘E20’, this area has not been included in the analysis.) We then rank the postcodes according to mean residential property price, where a rank of 1 indicates the highest mean residential property price. Finally, we calculate the relative changes in property price by determining the difference between the 2013 rank and the 2004 rank for each Inner London postcode. Thus, negative changes in rank signify an area becoming relatively more expensive, and positive changes in rank signify an area becoming relatively less expensive.

We note that other measures of change in house price are strongly correlated with the average house prices in an area. For example, areas with the highest mean house price are those with the highest change in mean house price. This is not true for change in rank mean house price. An alternative approach to resolving this issue would be to normalize changes in mean house price, by dividing the change by the original mean house price for an area.

## 3. Results

**Figure 2.**
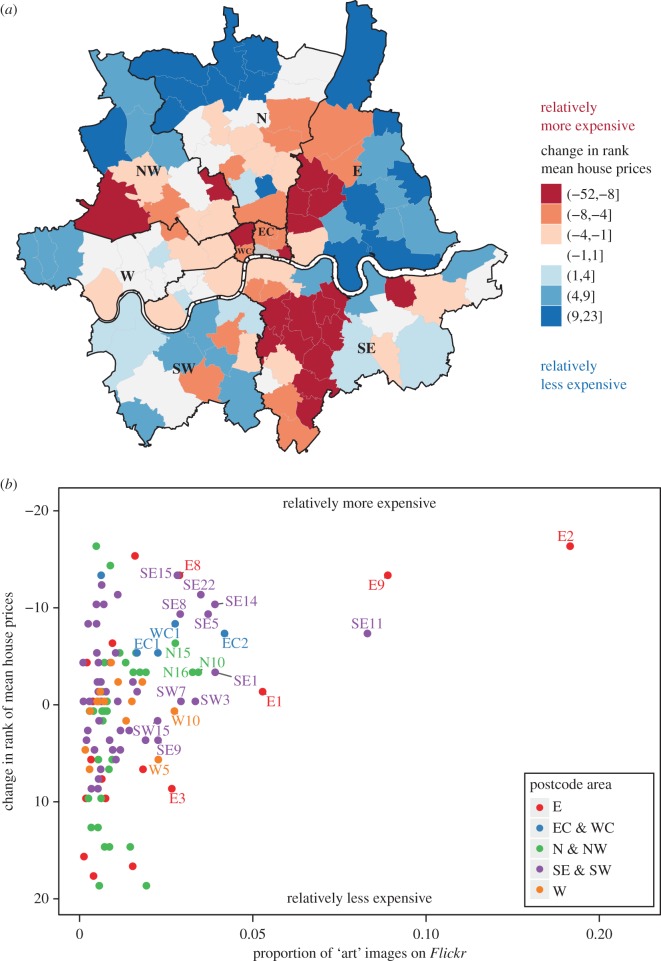
Relative change of mean residential property prices and the relation to ‘art’ photographs in Inner London postcode areas. (*a*) Areas in the eastern part of Central London and Southeast London have become relatively more expensive while areas in East and North London have become relatively less expensive. Property prices are tracked using data from residential property sales registered with the Land Registry from 2004 to 2013. Mean residential property prices per Inner London neighbourhood are ranked, where 1 is the highest mean residential property price. The change in rank is calculated over the entire time period, and, therefore, represents the 2013 rank minus the 2004 rank. Negative changes in rank (highlighted in red) signify areas becoming relatively more expensive, while positive changes in rank (highlighted in blue) signify areas becoming relatively less expensive. Postal boundaries © GeoLytix copyright and database right 2012. Contains Ordnance Survey data © Crown copyright and database right 2012. Contains Royal Mail data © Royal Mail copyright and database right 2012. Contains National Statistics data © Crown copyright and database right 2012. (*b*) A comparison of the proportion of ‘art’ related photographs for each Inner London postcode area and the change in rank of mean residential property prices shows that such areas have greater relative gains in residential property prices. (Note that the axis for the change in rank of mean residential property prices is reversed.) Areas thought to be associated with art-led economic development such as Shoreditch (E2) and Dalston (E9) clearly stand out as having risen in rank in terms of house prices, as well as containing a high proportion of ‘art’-related photographs. We see that the same observation can be made about a number of areas that are not frequently discussed in the media in terms of art-led gentrification, such as Vauxhall (SE11), Lewisham (SE8) and Lambeth (SE5).

Visual inspection of the change in rank of mean residential property prices reveals that areas in central East and Southeast London have become relatively more expensive while areas in outer East London and North London have become relatively less expensive (figure 2*a*). A comparison of the relative change of mean residential property price and the proportion of ‘art’ images suggests that the higher the proportion of ‘art’ images, the greater the relative gain in house price, as measured by the change in rank of mean residential property prices (figure 2*b*). A Kendall rank correlation test provides further evidence of this relationship (*τ* = −0.23, *p* < 0.001, *n* = 119, Kendall's rank correlation). This is initial evidence in support of our hypothesis that the presence of art is associated with improvements in economic conditions of urban neighbourhoods.

However, in general, measurements of social and physical characteristics can be clustered in geographical space, and thus spatially dependent, where nearby observations may have similar values simply due to their proximity [36,37]. We test whether such spatial clustering exists by first building a linear regression model to predict relative change in mean residential property price from the proportion of ‘art’ images. We determine which postcode areas are neighbours based on whether they share any boundary points, using boundary-line data for Great Britain from the Ordinance Survey (https://www.ordnancesurvey.co.uk/opendatadownload/products.html). Neighbours are defined in a binary fashion: either two areas are neighbours, or they are not. Using this neighbour structure, we confirm that our model contains measurements clustered in space by running a Moran's *I* test on the residuals of the linear regression model (Moran's *I *= 0.281, *p* < 0.001, *n* = 119).

The presence of clustering in the data means that we cannot guarantee that our observations are independent, a key requirement of linear regression. In order to address the problem of spatial autocorrelation in the residuals of our basic linear regression model, we build a conditional auto regressive (CAR) model. The CAR model is a linear regression model which additionally captures the spatial dependency between the error term for each area and the error terms for the neighbouring areas. Further details of this model can be found in the electronic supplementary material.

This CAR model is used to predict relative change in mean residential property price using the proportion of ‘art’ images. We find that areas with higher proportions of ‘art’ images also have greater relative gains in house price, as measured by the change in rank of mean residential property prices (*β *= −90.92, *p *= 0.040, *n *= 119).

We note that a small number of areas, such as Shoreditch (E2) and Dalston (E9) which are commonly thought to be associated with arts-led redevelopment, exhibit particularly high proportions of ‘art’-related photographs, as well as high relative gains in house price (figure 2*b*). We also find that the residuals from our model are not normally distributed and exhibit a slight skew. To ensure that the presence of this handful of cases and the distributional properties of our residuals are not driving our results, we run a bootstrap analysis on our CAR model [38], in which we compare the original regression coefficient with a bootstrapped distribution of regression coefficients.

To carry out this bootstrap analysis, we resample the residuals of our CAR model 10 000 times. We use these bootstrapped residuals to generate bootstrapped changes in rank of mean residential property prices. Finally, we build CAR models to regress the bootstrapped changes in property price on the proportions of ‘art’ photographs in each area, which remain the same as in the original CAR model, in order to create 10 000 bootstrapped regression coefficients. A comparison of the original regression coefficient with the bootstrapped distribution of regression coefficients provides further evidence that areas which have a higher proportion of art images have greater relative gains in house price, as measured by the change in rank of mean residential property prices (mean bootstrapped *β *= −116.74, 95% confidence interval [−209.85, −32.19], *p* = 0.044, 10 000 bootstrap samples).

## 4. Conclusion

We consider whether geotagged photographs uploaded to *Flickr* may help us quantify the link between art and the changing economic conditions of urban neighbourhoods. We quantify art in a neighbourhood using the proportion of ‘art’ photographs uploaded to *Flickr*. We compare our art indicator with the relative change in mean residential property price for each Inner London neighbourhood. Once we take clustering of spatial measurements into account, relative increases in mean residential property prices are significantly associated with higher proportions of ‘art’ images per neighbourhood.

The nature, as well as the direction, of any causal link requires more investigation. Does the presence of art in an area remove a negative stigma attached to deprived neighbourhoods [9]? Do artists improve the aesthetics of deprived neighbourhoods through the creation of public artworks such as iconic sculptures and street murals [39–41]? Or, as people connected to the arts rely on socializing to advance their careers, are more ‘arty’ neighbourhoods attracting more cafes and restaurants, which in turn attract other groups of people to move into the neighbourhood [1,10]? We highlight once more that it is also possible that ‘self-selection’ may be influencing our measure of art in a neighbourhood. As property prices increase, such neighbourhoods might attract more affluent people with interests in art, who might then be more likely to search for art in their neighbourhood which they can photograph and display on *Flickr*.

We also underline that the study we present here considers only one aspect of urban economic change. Future studies may wish to extend this analysis to other related socio-economic variables. For example, as areas regenerate, artists themselves may be displaced [8,42,43]. Studies quantifying the presence of art over time alongside changing socio-economic factors could be vital in better understanding this process. Finally, the link between the presence of art and increasing property prices might be due to a combination of factors. It has previously been argued that in neighbourhoods where a restricted supply of housing is not pressuring property prices, artist-led economic development may not take place [11,44]. Caution should, therefore, be taken in singling out art alone as the driving force behind improving economic conditions of urban neighbourhoods.

Our results suggest that art is indeed associated with improving economic conditions of urban neighbourhoods. More generally, our analysis demonstrates that data on our online interactions can provide novel measurements of the environment in which we live. These measurements reveal that the visual environment may affect aspects of our life as crucial as economic development.

## Supplementary Material

Supplementary Material - Further Methods and Data Retrieval

## Data accessibility

Datasets used in this study are available via the Dryad Repository (http://dx.doi.org/10.5061/dryad.6q5vf).

## References

[1] Currid E 2007 The Warhol economy: how fashion, art, and music drive New York City. Princeton, NJ: Princeton University Press.

[2] Florida RL 2002 The rise of the creative class: and how it*’*s transforming work, leisure, community and everyday life. New York, NY: Basic Books.

[3] Markusen A, Gadwa A 2010 Arts and culture in urban or regional planning: a review and research agenda. J. Plann. Educ. Res. 29, 379–391. (doi:10.1177/0739456X09354380)

[4] Nicodemus AG 2013 Fuzzy vibrancy: creative placemaking as ascendant US cultural policy. Cult. Trends 22, 213–222. (doi:10.1080/09548963.2013.817653)

[5] Vicario L, Martinez Monje PM 2003 Another ‘Guggenheim effect’? The generation of a potentially gentrifiable neighbourhood in Bilbao. Urban Stud. 40, 2383–2400. (doi:10.1080/0042098032000136129)

[6] Cameron S, Coaffee J 2005 Art, gentrification and regeneration: from artist as pioneer to public arts. Eur. J. Housing Policy 5, 39–58. (doi:10.1080/14616710500055687)

[7] Cole DB 1987 Artists and urban redevelopment. Geogr. Rev. 77, 391–407. (doi:10.2307/214280)

[8] Ley D 2003 Artists, aestheticisation and the field of gentrification. Urban Stud. 40, 2527–2544. (doi:10.1080/0042098032000136192)

[9] Lloyd R 2002 Neo–Bohemia: art and neighborhood redevelopment in Chicago. J. Urban Aff. 24, 517–532. (doi:10.1111/1467-9906.00141)

[10] Molotch H, Treskon M 2009 Changing art: SoHo, Chelsea and the dynamic geography of galleries in New York City. Int. J. Urban Reg. Res. 33, 517–541. (doi:10.1111/j.1468-2427.2009.00866.x)

[11] Markusen A 2006 Urban development and the politics of a creative class: evidence from a study of artists. Environ. Plann. A 38, 1921–1940. (doi:10.1068/a38179)

[12] Sands G, Reese LA 2013 Fair weather friends? The impact of the creative class on the economic health of mid-sized US metropolitan areas, 1990–2009. Camb. J. Regions Econ. Soc. 6, 71–91. (doi:10.1093/cjres/rss013)

[13] Grodach C, Foster N, Murdoch J 2014 Gentrification and the artistic dividend: the role of the arts in neighborhood change. J. Am. Plann. Assoc. 80, 21–35. (doi:10.1080/01944363.2014.928584)

[14] Schuetz J 2012 Causal agents or canaries in the coal mine? Art galleries and neighborhood change. In Creative communities: art works in economic development (ed. Rushton M), pp. 12–35. Washington, DC: Brookings Institution Press.

[15] Schuetz J 2014 Do art galleries stimulate redevelopment? J. Urban Econ. 83, 59–72. (doi:10.1016/j.jue.2014.08.002)

[16] Foster N, Grodach C, Murdoch J In press Neighborhood diversity, economic health, and the role of the arts. J. Urban Aff. (doi:10.1111/juaf.12278)

[17] Alanyali M, Preis T, Moat HS 2016 Tracking protests using geotagged Flickr photographs. PLoS ONE 11, e0150466 (doi:10.1371/journal.pone.0150466) 26930654 10.1371/journal.pone.0150466 PMC4773018

[18] Barchiesi D, Moat HS, Alis C, Bishop S, Preis T 2015 Quantifying international travel flows using Flickr. PLoS ONE 10, e0128470 (doi:10.1371/journal.pone.0128470) 26147500 10.1371/journal.pone.0128470 PMC4493158

[19] Batty M 2013 Big data, smart cities and city planning. Dialogues Hum. Geogr. 3, 274–279. (doi:10.1177/2043820613513390) 10.1177/2043820613513390 PMC5808818 29472982

[20] Botta F, Moat HS, Preis T 2015 Quantifying crowd size with mobile phone and Twitter data. R. Soc. open sci. 2, 150162 (doi:10.1098/rsos.150162) 26064667 10.1098/rsos.150162 PMC4453255

[21] Choi H, Varian H 2012 Predicting the present with Google Trends. Econ. Rec. 88, 2–9. (doi:10.1111/j.1475-4932.2012.00809.x)

[22] Graham M, Shelton T 2013 Geography and the future of big data, big data and the future of geography. Dialogues Hum. Geogr. 3, 255–261. (doi:10.1177/2043820613513121)

[23] King G 2011 Ensuring the data-rich future of the social sciences. Science 331, 719–721. (doi:10.1126/science.1197872) 21311013 10.1126/science.1197872

[24] Lazer D et al. 2009 Computational social science. Science 323, 721–723. (doi:10.1126/science.1167742) 19197046 10.1126/science.1167742 PMC2745217

[25] Letchford A, Preis T, Moat HS 2016 Quantifying the search behaviour of different demographics using Google Correlate. PLoS ONE 11, e0149025 (doi:10.1371/journal.pone.0149025) 26910464 10.1371/journal.pone.0149025 PMC4766235

[26] Moat HS, Curme C, Avakian A, Kenett DY, Stanley HE, Preis T 2013 Quantifying Wikipedia usage patterns before stock market moves. Sci. Rep. 3, 1801 (doi:10.1038/srep01801)

[27] Moat HS, Preis T, Olivola CY, Liu C, Chater N 2014 Using big data to predict collective behavior in the real world. Behav. Brain Sci. 37, 92–93. (doi:10.1017/S0140525X13001817) 24572233 10.1017/S0140525X13001817

[28] Preis T, Moat HS, Bishop SR, Treleaven P, Stanley HE 2013 Quantifying the digital traces of Hurricane Sandy on Flickr. Sci. Rep. 3, 3141 (doi:10.1038/srep03141) 24189490 10.1038/srep03141 PMC3817451

[29] Preis T, Moat HS, Stanley HE 2013 Quantifying trading behavior in financial markets using Google Trends. Sci. Rep. 3, 1684 (doi:10.1038/srep01684) 23619126 10.1038/srep01684 PMC3635219

[30] Seresinhe CI, Preis T, Moat HS 2015 Quantifying the impact of scenic environments on health. Sci. Rep. 5, 16899 (doi:10.1038/srep16899) 26603464 10.1038/srep16899 PMC4658473

[31] Vespignani A 2009 Predicting the behavior of techno-social systems. Science 325, 425–428. (doi:10.1126/science.1171990) 19628859 10.1126/science.1171990

[32] Zaltz Austwick M, O'Brien O, Strano E, Viana M 2013 The structure of spatial networks and communities in bicycle sharing systems. PLoS ONE 8, e74685 (doi:10.1371/journal.pone.0074685) 24040320 10.1371/journal.pone.0074685 PMC3765359

[33] The Economist. 2000 *The geography of cool.* See http://www.economist.com/node/303114 (accessed 5 May 2015).

[34] Crowley EJ, Zisserman A 2014 In search of art. Computer vision—ECCV 2014 workshops, pp. 54–70.

[35] Lintott CJ et al. 2008 Galaxy Zoo: morphologies derived from visual inspection of galaxies from the Sloan Digital Sky Survey. Mon. Not. R. Astron. Soc. 389, 1179–1199. (doi:10.1111/j.1365-2966.2008.13689.x)

[36] Bivand RS, Pebesma E, Gómez-Rubio V 2013 Applied spatial data analysis with R. New York, NY: Springer.

[37] Harris R, Sleight P, Webber R 2005 Geodemographics, GIS, and neighbourhood targeting. Chichester, UK: Wiley.

[38] Efron B, Tibshirani RJ 1994 An introduction to the Bootstrap. Boca Raton, FL: CRC Press.

[39] Deutsche R, Ryan CG 1984 The fine art of gentrification. October 31, 111–191. (doi:10.2307/778358)

[40] Grodach C 2010 Art spaces, public space, and the link to community development. Commun. Dev. J. 45, 474–493. (doi:10.1093/cdj/bsp018)

[41] Mathews V 2010 Aestheticizing space: art, gentrification and the city. Geogr. Compass 4, 660–675. (doi:10.1111/j.1749-8198.2010.00331.x)

[42] Bowler A, McBurney B 1991 Gentrification and the Avant-Garde in New York's East Village: the good, the bad and the ugly. Theory Cult. Soc. 8, 49–77. (doi:10.1177/026327691008004003)

[43] Atkinson R 2000 The hidden costs of gentrification: displacement in central London. J. Housing Built Environ. 15, 326–307. (doi:10.1023/A:1010128901782)

[44] Stern M 2003 *Culture and the changing urban landscape: Philadelphia 1997–2002*. In Paper presented at the Regional Science Association International meetings, 22 November. Philadelphia, PA: RSAI.

