# Supplementary material for: Quantifying the link between art and property prices in urban neighbourhoods
Source: R Soc Open Sci. 2016 Apr 27;3(4):160146. doi: 10.1098/rsos.160146 (PMC4852651; doi:10.1098/rsos.160146)
Supplement: Supplementary Material - Further Methods and Data Retrieval [file rsos160146supp1.pdf]

TITLE:

Quantifying the Link Between Art and Property Prices in Urban Neighbourhoods

Supplementary Material

AUTHORS:

Chanuki Illushka Seresinhe[1*], Tobias Preis[1] and Helen Susannah Moat[1]

AFFILIATIONS:

[1] Data Science Lab, Behavioural Science, Warwick Business School,

University of Warwick, Coventry, CV4 7AL, UK

* To whom correspondence should be addressed:

E-mail: C.Seresinhe@warwick.ac.uk

SUPPLEMENTARY MATERIAL:

Spatial analysis and mapping

All statistical analysis was carried out using R. Spatial statistics were produced using a combination of spatial statistics libraries including the 'sp' package [1,2], which includes classes and methods for spatial data, and 'spdep' [3,4] for spatial dependent weighting schemes. The primary library used to match spatial data between different geographies and plot maps was 'rgdal' [5], which enables R to read shapefiles into spatial vector objects and access the following spatial libraries: GDAL (Geospatial Data Abstraction Library) and Proj.4 (Cartographic Projections Library). Map graphics have been created using boundary data retrieved from the UK Data Service [6] and the R package 'ggplot' [7].

Conditional Auto Regressive (CAR) model

When working with spatial data, it is reasonable to assume that observations in neighbouring areas may be more or less alike simply due to their proximity and hence exhibit autocorrelation [1, 8]. We confirm this by first running a Moran's I test, which measures whether spatial autocorrelation is present in the data. Due to this autocorrelation, we cannot run a simple linear regression analysis, as spatial dependencies would exist in the error term. Hence, we run our analysis using a conditional autoregressive prior (CAR), as initially proposed by Besag and colleagues [9,10], which captures spatial dependence between neighbours through an adjacency matrix of the areal units.

The CAR model quantifies the spatial relationship in the data by including a conditional distribution in the error term for area i, e_i. The conditional distribution of e_i is thus represented as:

$$e_i | e_{j\sim i} \sim N\left(\sum_{j\sim i} \frac{c_{ij} e_j}{\sum_{j\sim i} c_{ij}}, \frac{\sigma_{e_i}^2}{\sum_{j\sim i} c_{ij}}\right) \tag{1}$$

where $e_{j\sim i}$ is the e_{-i} vector including only neighbouring areas of i; e_{-i} is the vector of all the errors terms except for itself; and c_{ij} are dependence parameters used to represent the spatial dependence between the areas.

Data retrieval

Data on *Flickr* images were retrieved from the *Flickr* Application Programming Interface (https://www.flickr.com/services/api/) in December 2013. Data on residential property sales were produced by Land Registry © Crown copyright 2015. Residential property sales from 2004 to 2013 were retrieved on 19 December 2014 from https://www.gov.uk/government/collections/price-paid-data.

SUPPLEMENTARY MATERIAL REFERENCES:

1. Bivand, R. S., Pebesma, E., & Gómez-Rubio, V. 2013 *Applied Spatial Data Analysis with R*. New York: Springer New York.
2. Pebesma, E.J., Bivand, R.S. 2005 Classes and methods for spatial data in R. *R News* **5,** 2 (http://cran.r-project.org/doc/Rnews/).
3. Bivand, R.S., & Piras, G. 2015 Comparing Implementations of Estimation. Methods for Spatial Econometrics. *Journal of Statistical Software*, **63**, 18 (http://www.jstatsoft.org/v63/i18/).
4. Bivand, R. S., Hauke, J., & Kossowski, T. 2013 Computing the Jacobian in Gaussian spatial autoregressive models: An illustrated comparison of available methods. *Geographical Analysis*, **45** 2
5. Bivand, R. S., Keitt, T. and Rowlingson, B. 2015 *rgdal: Bindings for the Geospatial Data Abstraction Library. R package version 1.0-4* (http://CRAN.R-project.org/package=rgdal)
6. GeoLytix 2012. *Boundary data (Great Britain)* [data collection]. UK Data Service. SN:5819 UKBORDERS: Digitised Boundary Data, 1840- and Postcode Directories, 1980-. http://discover.ukdataservice.ac.uk/catalogue/?sn=5819&type=Data%20catalogue, Retrieved from http://census.ukdataservice.ac.uk/get-data/boundary-data.aspx. Contains public sector information licensed under the Open Government Licence v1.
7. Wickham. H. 2009 *ggplot2: Elegant Graphics for Data Analysis.* New York: Springer-Verlag

8. Harris, R., P. Sleight, & Webber , R. 2005 *Geodemographics, GIS, and neighbourhood targeting*. Chichester: Wiley.

9. Besag, J., J. York, & Mollié, A. 1991 Bayesian image restoration, with two applications in spatial statistics. *Annals of the Institute of Statistical Mathematics* **43**, 1-20.

10. Besag, J. 1974 Spatial Interaction and the Statistical Analysis of Lattice Systems. *Journal of the Royal Statistical Society, Series B* **36**, 192-236.
